# Supplementary material for: Activation of Nkx2.5 transcriptional program is required for adult myocardial repair
Source: Nat Commun. 2022 May 27;13:2970. doi: 10.1038/s41467-022-30468-4 (PMC9142600; doi:10.1038/s41467-022-30468-4)
Supplement: Supplementary file 1 — Supplementary Information [file 41467_2022_30468_MOESM1_ESM.pdf]

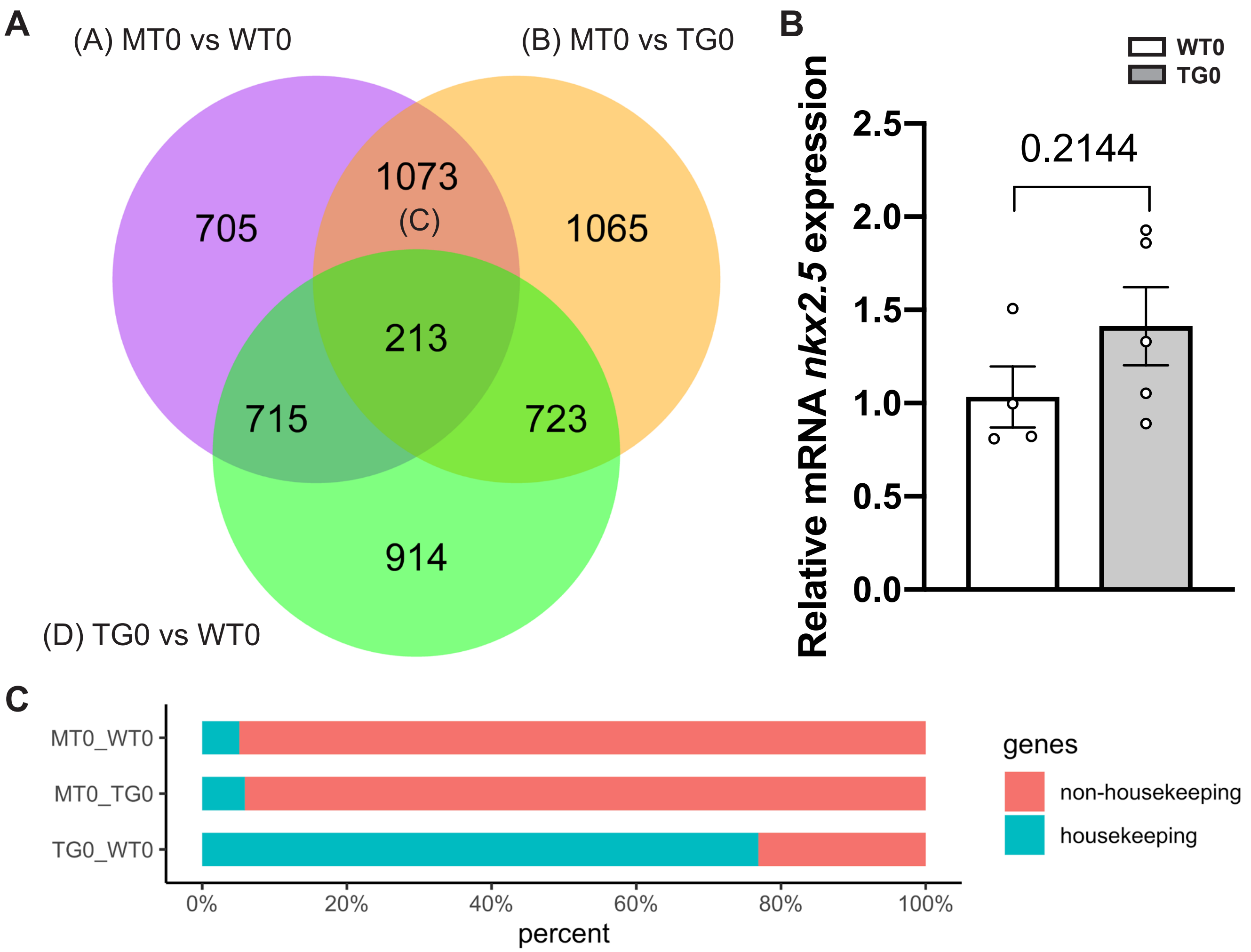
A
(A) MT0 vs WT0
(B) MT0 vs TG0
705
1073
(C)
1065
213
715
723
914
(D) TG0 vs WT0
B
WT0
TG0
Relative mRNA nkx2.5 expression
0.2144
2.5
2.0
1.5
1.0
0.5
0.0
C
MT0_WT0
MT0_TG0
TG0_WT0
0%
20%
40%
60%
80%
100%
percent
genes
non-housekeeping
housekeeping

Figure S1. 'Overlap' intersection category distinguishes critical differentially expressed genes due to the loss of Nkx2.5 function

(**A**) Venn overlap of DEGs (FC > 0.5; FDR < 0.05) in *nkx2.5*$^{-/-}$ compared to non-transgenic wild-type (group A, MT0 vs WT0) and transgenic wild-type (group B, MT0 vs TG0). These two sets are overlapped with a comparison between transgenic and non-transgenic wild-type backgrounds (group D, TG0 vs WT0).

(**B**) Relative *nkx2.5* expression as measured by qPCR comparing uninjured non-transgenic (n = 4) and transgenic (n = 5) wild-type hearts depicts no statistically significant difference in *Tg(hsp70l:nkx2.5-EGFP)* expression. Mean and standard error of each data set are shown with no statistically significant difference (p = 0.2144) identified by an unpaired, two-tailed t-test performed between WT0 and TG0.

(**C**) Percentage of 'housekeeping' genes in each dataset represented in Venn diagram.

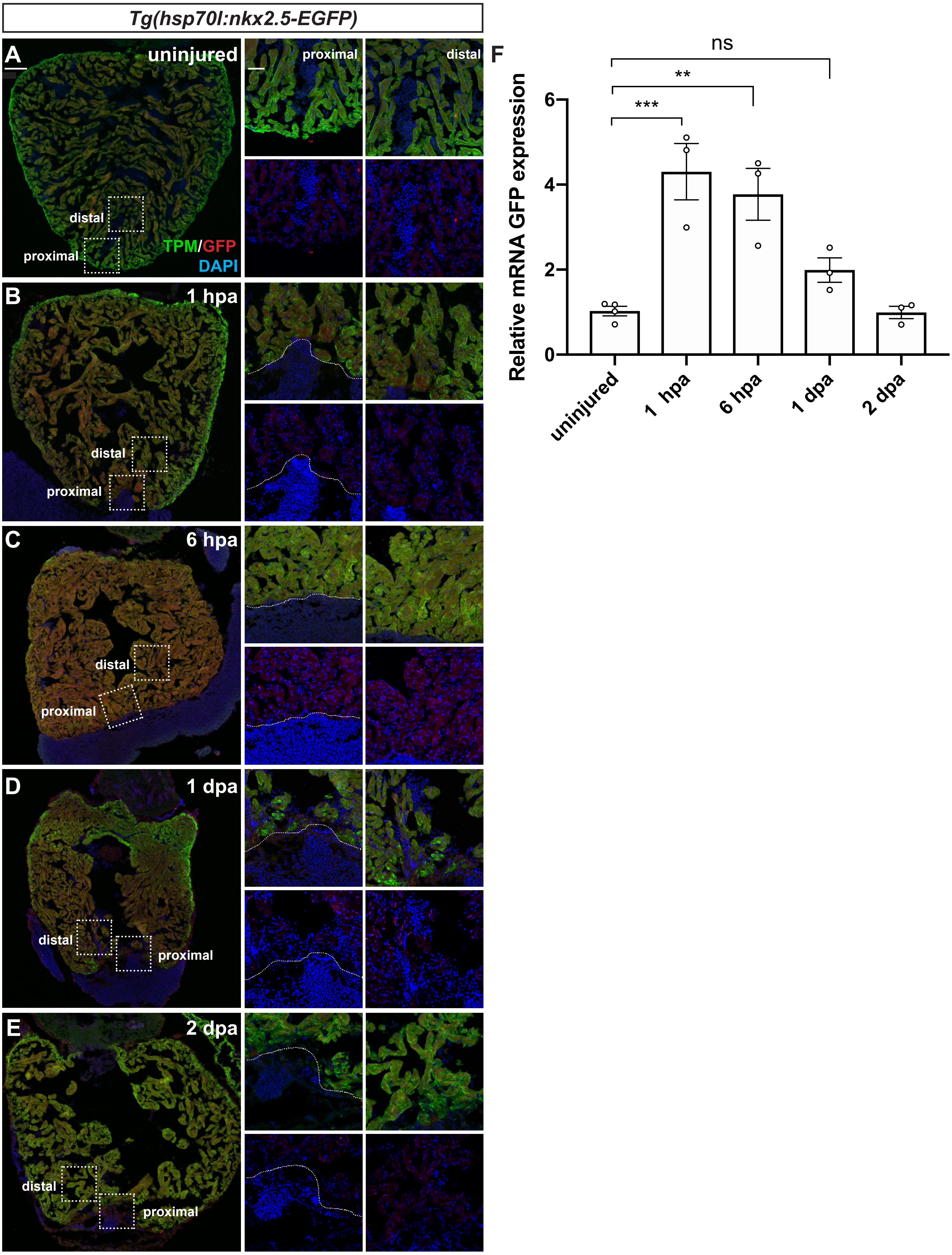

Tg(hsp70l:nkx2.5-EGFP)
A
uninjured
proximal
distal
TPM/GFP
DAPI
B
1 hpa
C
6 hpa
D
1 dpa
E
2 dpa
F
ns
**
***
Relative mRNA GFP expression
0
2
4
6
uninjured
1 hpa
6 hpa
1 dpa
2 dpa

Figure S2. Heat shock inducible transgene responds to amputation injury

(**A-E**) Tropomyosin immunostaining with anti-GFP antibody on sections of uninjured (n = 5) (A) and injured *Tg(hsp70l:nkx2.5-EGFP)* hearts at one hour post amputation (1 hpa) (n = 5) (B), 6 hpa (n = 5) (C), 1 dpa (n = 5) (D), and 2 dpa (n = 5) (E) demonstrates maximal expression of Nkx2.5-EGFP at 6 hpa with diminution of the protein by 2 dpa. Scale bar, 100 μm. Higher magnification panels indicate regions outlined in boxes and show proximal and distal Nkx2.5-EGFP expression in comparison to the injury site. Scale bar, 30 μm.

(**F**) Relative GFP expression as measured by qPCR comparing the uninjured and injured transgenic wild-type hearts demonstrating peak expression of *Tg(hsp70l:nkx2.5-EGFP)* at 1 hpa and normalization to endogenous levels by 2 dpa. Mean and standard error of each data set are shown. One-way ANOVA was performed, followed by Dunnett's test, to detect adjusted p values (0.0003, 0.0014, 0.2969 and >0.9999) comparing uninjured (n = 4) to 1 hpa (n = 3), 6 hpa (n = 3), 1 dpa (n = 3), and 2 dpa (n = 3), respectively.

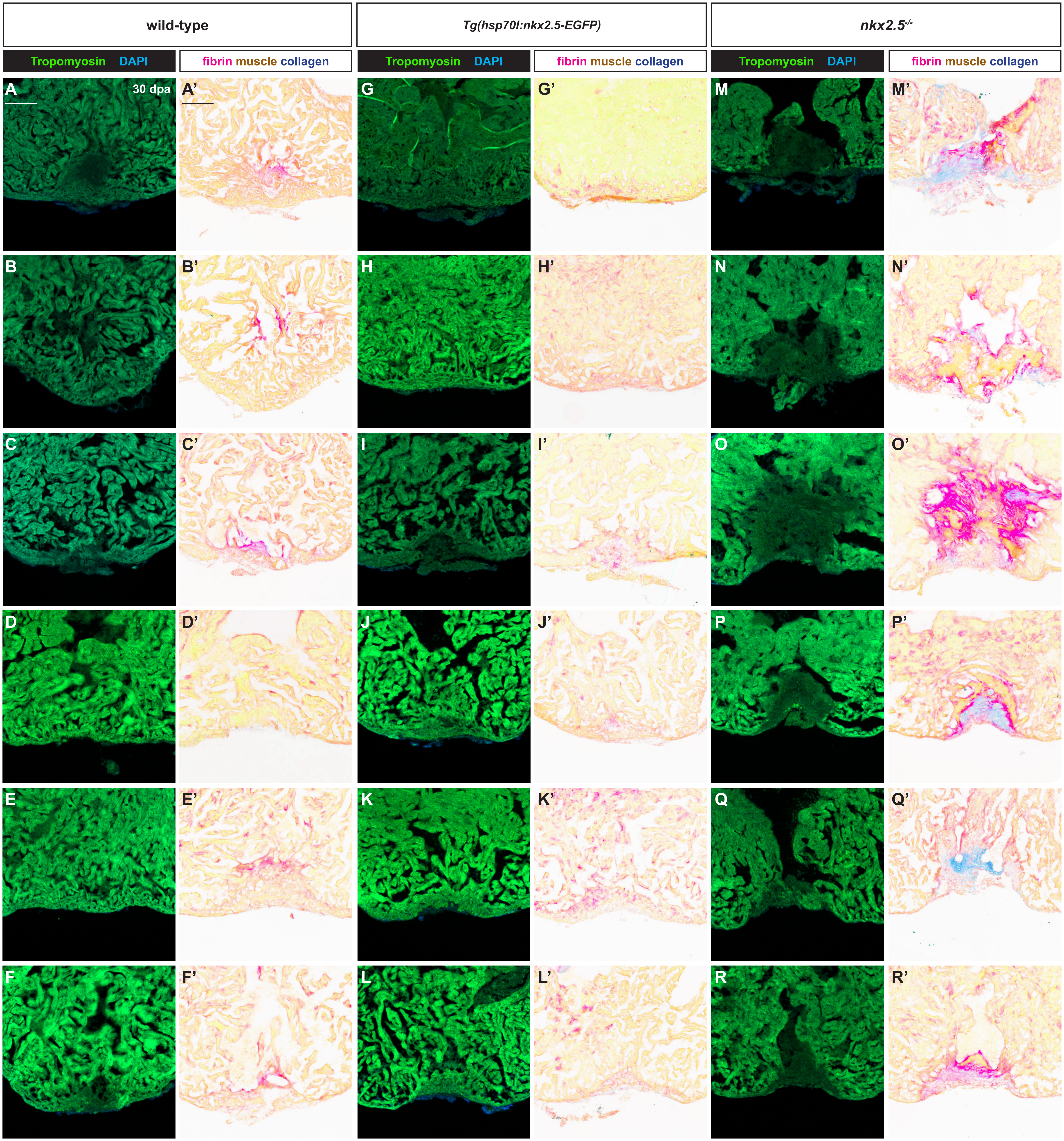

wild-type
Tg(hsp70l:nkx2.5-EGFP)
nkx2.5-/-
Tropomyosin DAPI
fibrin muscle collagen
Tropomyosin DAPI
fibrin muscle collagen
Tropomyosin DAPI
fibrin muscle collagen
A
30 dpa
A'
G
G'
M
M'
B
B'
H
H'
N
N'
C
C'
I
I'
O
O'
D
D'
J
J'
P
P'
E
E'
K
K'
Q
Q'
F
F'
L
L'
R
R'

Figure S3. Nkx2.5 is necessary for repair following myocardial injury

(**A-R'**) Representative sections from non-transgenic wild-type (n = 6) (A-F'), transgenic wild-type (n = 6) (G-L'), and *nkx2.5*$^{-/-}$ (n = 6) (M-R') hearts following Tropomyosin and AFOG staining at 30 dpa. Scale bar, 100 μm.

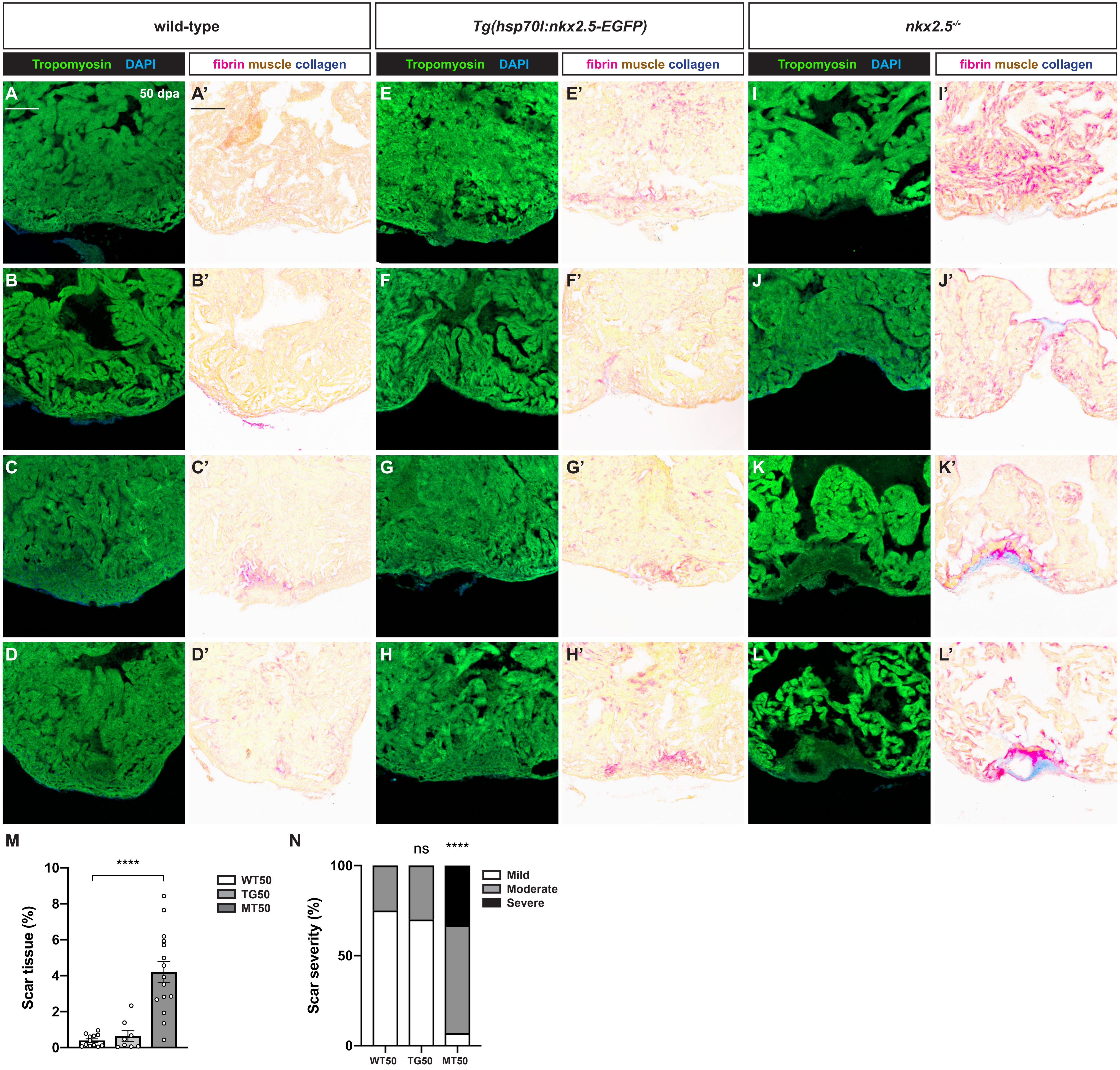

wild-type
Tg(hsp70l:nkx2.5-EGFP)
nkx2.5-/-
Tropomyosin DAPI
fibrin muscle collagen
Tropomyosin DAPI
fibrin muscle collagen
Tropomyosin DAPI
fibrin muscle collagen
A
50 dpa
A'
E
E'
I
I'
B
B'
F
F'
J
J'
C
C'
G
G'
K
K'
D
D'
H
H'
L
L'
M
****
Scar tissue (%)
10
8
6
4
2
0
WT50
TG50
MT50
N
ns
****
Scar severity (%)
100
50
0
Mild
Moderate
Severe
WT50
TG50
MT50

Figure S4. ***nkx2.5$^{-/-}$*** **fish fail to regenerate after ventricular apex resection**

(**A-L'**) Representative sections from non-transgenic wild-type (n =15) (A-D'), transgenic wild-type (n = 10) (E-H'), and *nkx2.5$^{-/-}$* hearts (n = 15) (I-L') hearts following Tropomyosin and AFOG staining at 50 dpa. Scale bar, 100 μm.

(**M,N**) Quantification of the percentage of scar tissue per ventricle (M) and the degree of scar severity (N) in non-transgenic wild-type (n = 12), transgenic wild-type (n = 9), and *nkx2.5$^{-/-}$* (n = 15) fish illustrates the significantly diminished reparative response in the absence of *nkx2.5* gene function. Mean and standard error of each data set are shown in (M). Unpaired, two-tailed t-tests were applied to show a statistically significant difference between WT50 and MT50 ($p < 0.0001$) and no statistically significant difference between WT50 and TG50 ($p = 0.4301$) in (M). Two-sided Fisher's exact test yields p value of 0.5267 comparing WT50 and TG50 and p value of < 0.0001 comparing WT50 and MT50 in (N).

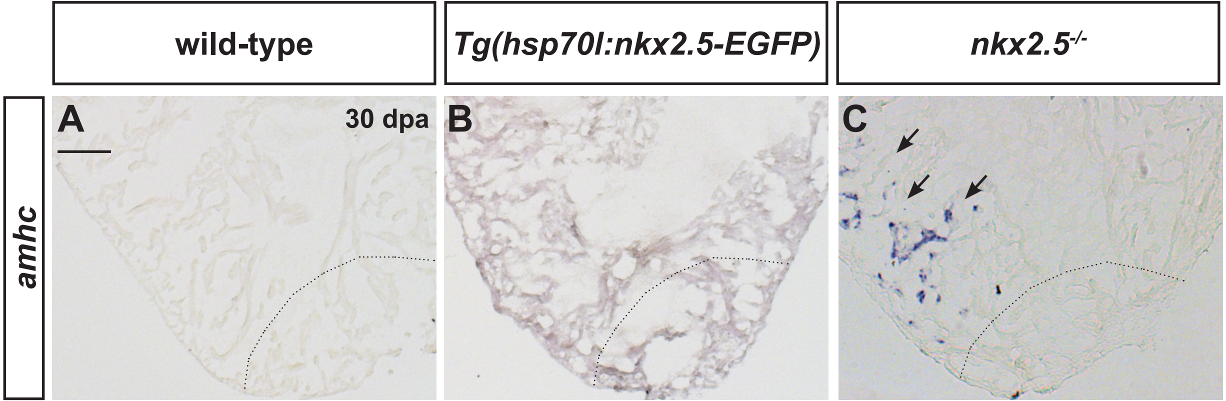

wild-type
Tg(hsp70l:nkx2.5-EGFP)
nkx2.5-/-
amhc
A
30 dpa
B
C

Figure S5. Chamber-specific identity is maintained in the regenerate in *nkx2.5*$^{-/-}$ hearts

(**A-C**) *In situ* hybridization reveals ectopic *amhc* expression in the ventricular myocardium of the *nkx2.5*$^{-/-}$ hearts (n = 6) (C), but not in the non-transgenic wild-type (n = 6) (A) and transgenic wild-type (n = 6) (B) hearts. Yet, ectopic *amhc* expression is excluded from the regenerate at 30 dpa (C). Dashed lines represent amputation planes. Scale bar, 100 μm.

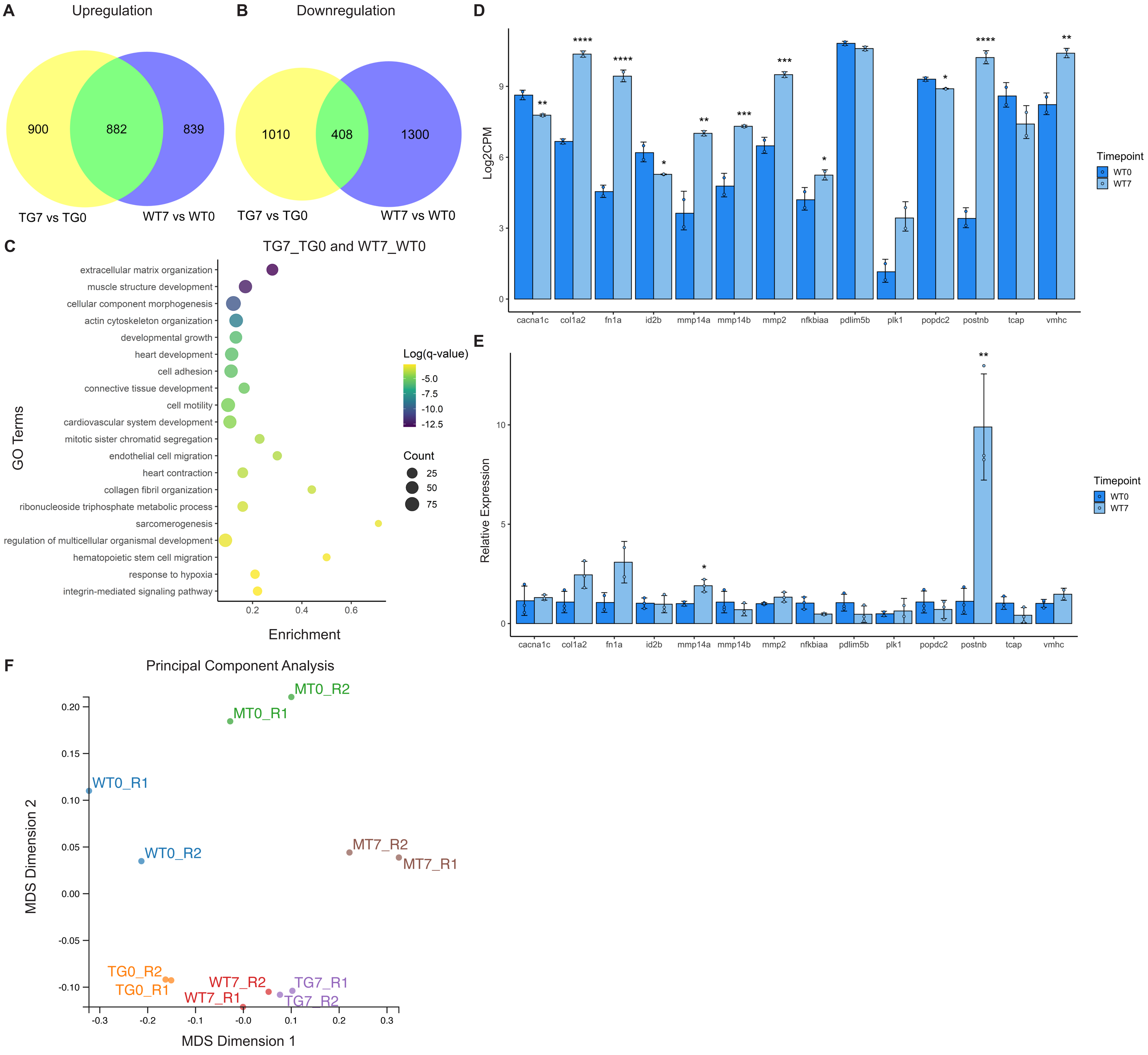

A
Upregulation
900
882
839
TG7 vs TG0
WT7 vs WT0
B
Downregulation
1010
408
1300
TG7 vs TG0
WT7 vs WT0
C
TG7_TG0 and WT7_WT0
GO Terms
extracellular matrix organization
muscle structure development
cellular component morphogenesis
actin cytoskeleton organization
developmental growth
heart development
cell adhesion
connective tissue development
cell motility
cardiovascular system development
mitotic sister chromatid segregation
endothelial cell migration
heart contraction
collagen fibril organization
ribonucleoside triphosphate metabolic process
sarcomerogenesis
regulation of multicellular organismal development
hematopoietic stem cell migration
response to hypoxia
integrin-mediated signaling pathway
Enrichment
Log(q-value)
Count
D
Log2CPM
Timepoint
WT0
WT7
cacna1c col1a2 fn1a id2b mmp14a mmp14b mmp2 nfkbiaa pdlim5b plk1 popdc2 postnb tcap vmhc
E
Relative Expression
Timepoint
WT0
WT7
F
Principal Component Analysis
MDS Dimension 1
MDS Dimension 2
MT0_R1
MT0_R2
WT0_R1
WT0_R2
MT7_R1
MT7_R2
TG0_R1
TG0_R2
WT7_R1
WT7_R2
TG7_R1
TG7_R2

Figure S6. Regenerative transcriptional profile is appropriately recruited in wild-type and *Tg(hsp70l:nkx2.5-EGFP)* fish

(**A,B**) Venn diagrams of DEGs (FC > 0.5; FDR < 0.05) overlapping between TG7 vs TG0 and WT7 vs WT0 comparisons. The 882 upregulated genes are represented in (A) and the 408 downregulated genes are represented in (B); both sets in green.

(**C**) GO analysis of the shared DEGs (green in A and B) are depicted; highly enriched categories such as extracellular matrix organization, actin cytoskeleton organization, and mitotic sister chromatid segregation suggest activation of normal regenerative mechanisms. Color and size of circles correspond to log (q-value) and enrichment, respectively.

(**D**) Log2 transformed RNA-seq mean counts per million (log2CPM) (± SD) of vital regenerative genes in WT7 vs WT0. Adjusted p values are obtained using Voom/Limma and demonstrate statistical difference for *cacna1c* (FDR = 0.0086), *col1a2* (FDR = 2.35E-06), *fn1a* (FDR = 1.14E-05), *id2b* (FDR = 0.0105), *mmp14a* (FDR = 0.0012), *mmp14b* (FDR = 0.0002), *mmp2* (FDR = 0.0004), *nfkbiaa* (FDR = 0.0171), *popdc2* (FDR = 0.0305), *postnb* (FDR = 2.89E-06) and *vmhc* (FDR = 0.0016) and no difference for *pdlim5b* (FDR = 0.1674), *plk1* (FDR = 0.1180), and *tcap* (FDR = 0.1068).

(**E**) Mean relative gene expression (± SD) of qPCR validated genes in WT7 vs WT0. Unpaired, two-tailed t-tests reveal statistically significant difference for *mmp14a* (p = 0.0334) and *postnb* (p = 0.0052) and no difference for *cacna1c* (p =

0.7311), *col1a2* (p = 0.0520), *fn1a* (p = 0.1315), *id2b* (p = 0.8762), *mmp14b* (p = 0.3492), *mmp2* (p = 0.0859), *nfkbiaa* (p = 0.0941), *pdlim5b* (p = 0.1646), *plk1* (p = 0.8481), *popdc2* (p = 0.4162), *tcap* (p = 0.0999), and *vmhc* (p = 0.1259).

(**F**) PCA plot shows clustering of RNA-seq samples with duplicates for each condition.

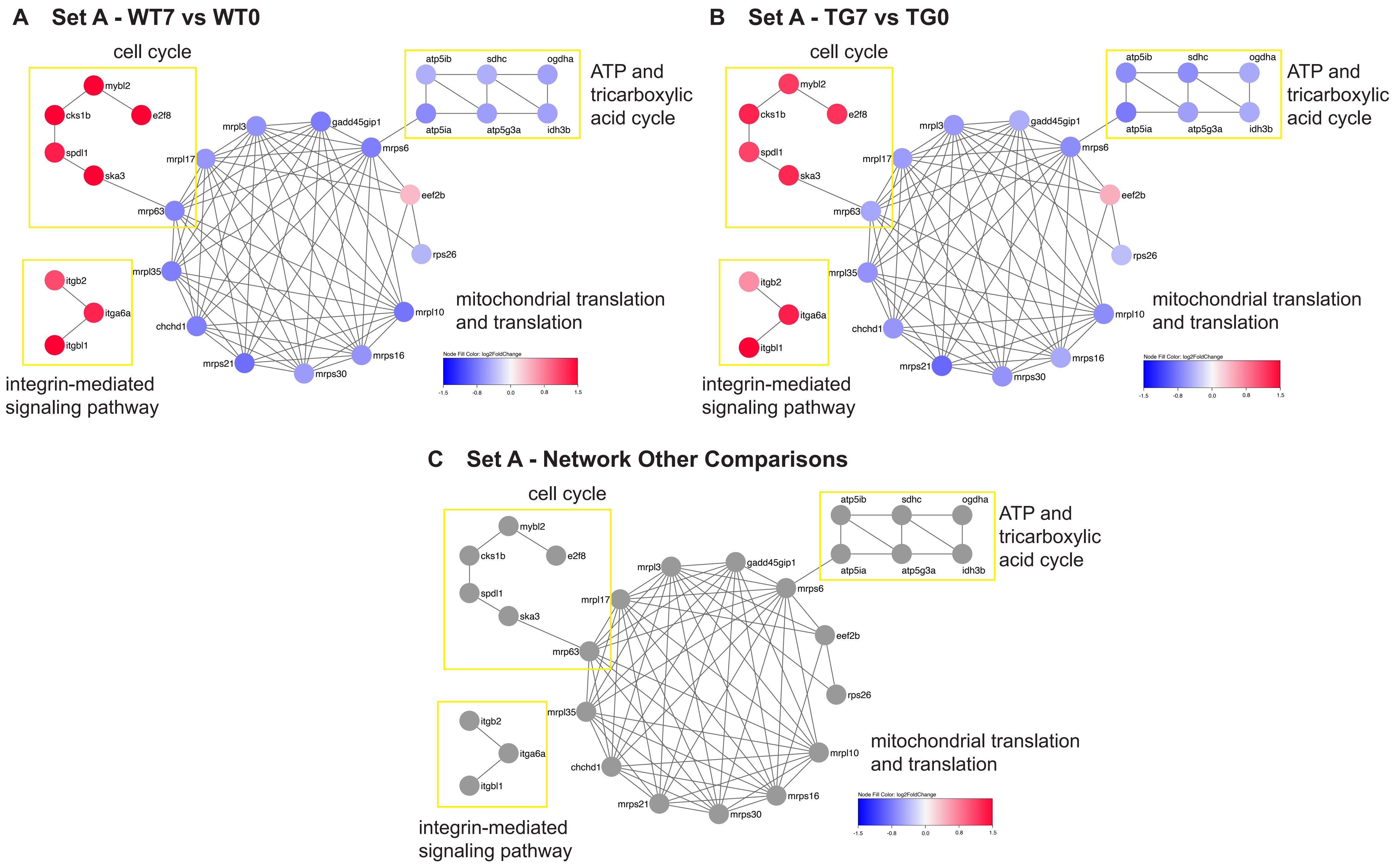
A Set A - WT7 vs WT0
B Set A - TG7 vs TG0
C Set A - Network Other Comparisons
cell cycle
mybl2
cks1b
e2f8
spdl1
ska3
mrp63
ATP and tricarboxylic acid cycle
atp5ib
sdhc
ogdha
atp5ia
atp5g3a
idh3b
mrpl3
gadd45gip1
mrps6
mrpl17
eef2b
rps26
mrpl35
mrpl10
chchd1
mrps16
mrps21
mrps30
mitochondrial translation and translation
integrin-mediated signaling pathway
itgb2
itga6a
itgbl1
Node Fill Color: log2FoldChange
-1.5
-0.8
0.0
0.8
1.5

Figure S7. Critical regenerative GRNs fail to be activated in *nkx2.5*$^{-/-}$ following injury

Network analyses of genes associated with significantly enriched GO terms from set A in Fig. 5A. Each comparison included and those comparisons that are excluded in the turquoise column of the UpSet Plot are depicted, emphasizing genes that are deployed in wild-type hearts following amputation but are not recruited in the absence of *nkx2.5* gene function. Red is upregulated and blue is downregulated, as shown in the keys.

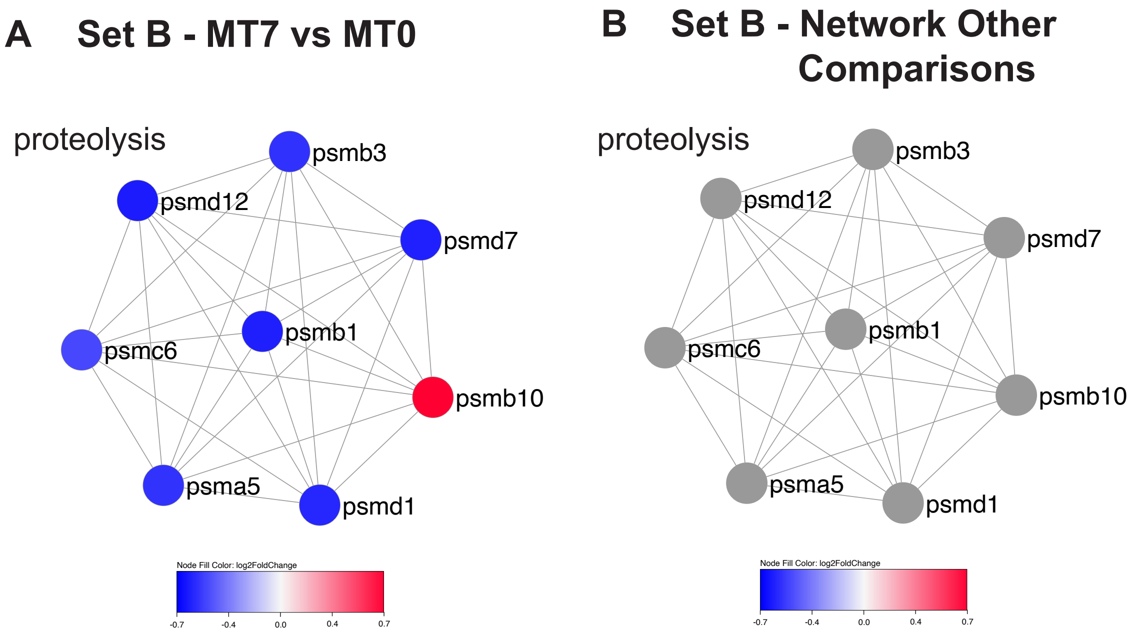
A
Set B - MT7 vs MT0
proteolysis
psmb3
psmd12
psmd7
psmb1
psmc6
psmb10
psma5
psmd1
Node Fill Color: log2FoldChange
-0.7
-0.4
0.0
0.4
0.7
B
Set B - Network Other Comparisons
proteolysis
psmb3
psmd12
psmd7
psmb1
psmc6
psmb10
psma5
psmd1
Node Fill Color: log2FoldChange
-0.7
-0.4
0.0
0.4
0.7

Figure S8. Proteolysis is uniquely suppressed in the injured *nkx2.5*$^{-/-}$ heart

Network analysis of genes associated with significantly enriched GO terms from set B (magenta column) in Fig. 5A accentuates downregulation of several, closely linked members of the *psmd* gene family, proteosome 26S subunit, non-ATPase in *nkx2.5*$^{-/-}$ fish specifically. Red is upregulated and blue is downregulated, as shown in the keys.

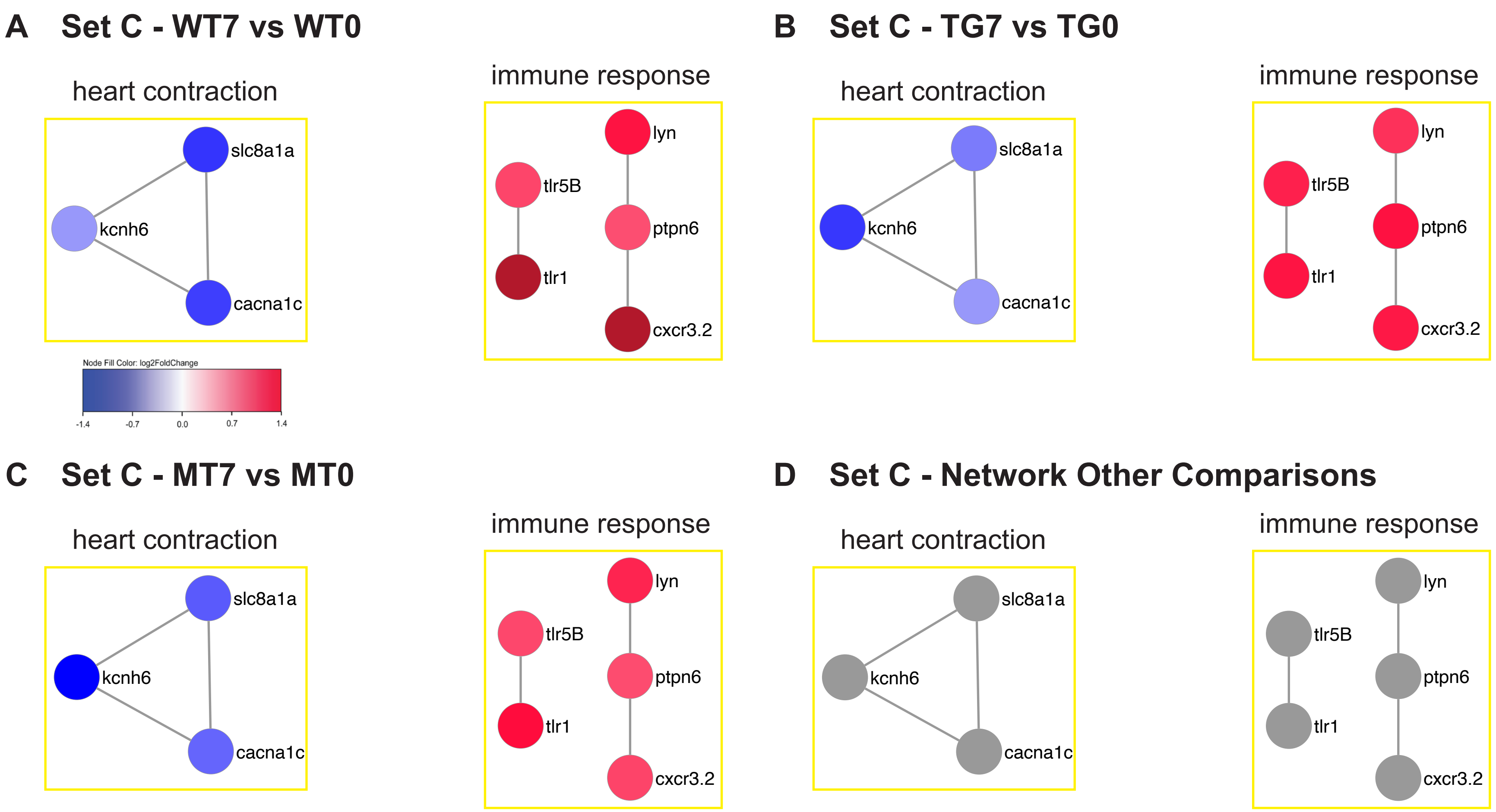
A Set C - WT7 vs WT0
heart contraction
slc8a1a
kcnh6
cacna1c
immune response
lyn
tlr5B
ptpn6
tlr1
cxcr3.2
Node Fill Color: log2FoldChange
-1.4
-0.7
0.0
0.7
1.4
B Set C - TG7 vs TG0
heart contraction
slc8a1a
kcnh6
cacna1c
immune response
lyn
tlr5B
ptpn6
tlr1
cxcr3.2
C Set C - MT7 vs MT0
heart contraction
slc8a1a
kcnh6
cacna1c
immune response
lyn
tlr5B
ptpn6
tlr1
cxcr3.2
D Set C - Network Other Comparisons
heart contraction
slc8a1a
kcnh6
cacna1c
immune response
lyn
tlr5B
ptpn6
tlr1
cxcr3.2

Figure S9. Heart contraction and immune response GRNs are appropriately engaged in the *nkx2.5* loss-of-function model

Set C (black column) in Fig. 5A embodies all DEGs that are enlisted in the *nkx2.5*$^{-/-}$ fish, consistent with wild-type regenerative mechanisms. Red is upregulated and blue is downregulated, as shown in the keys.

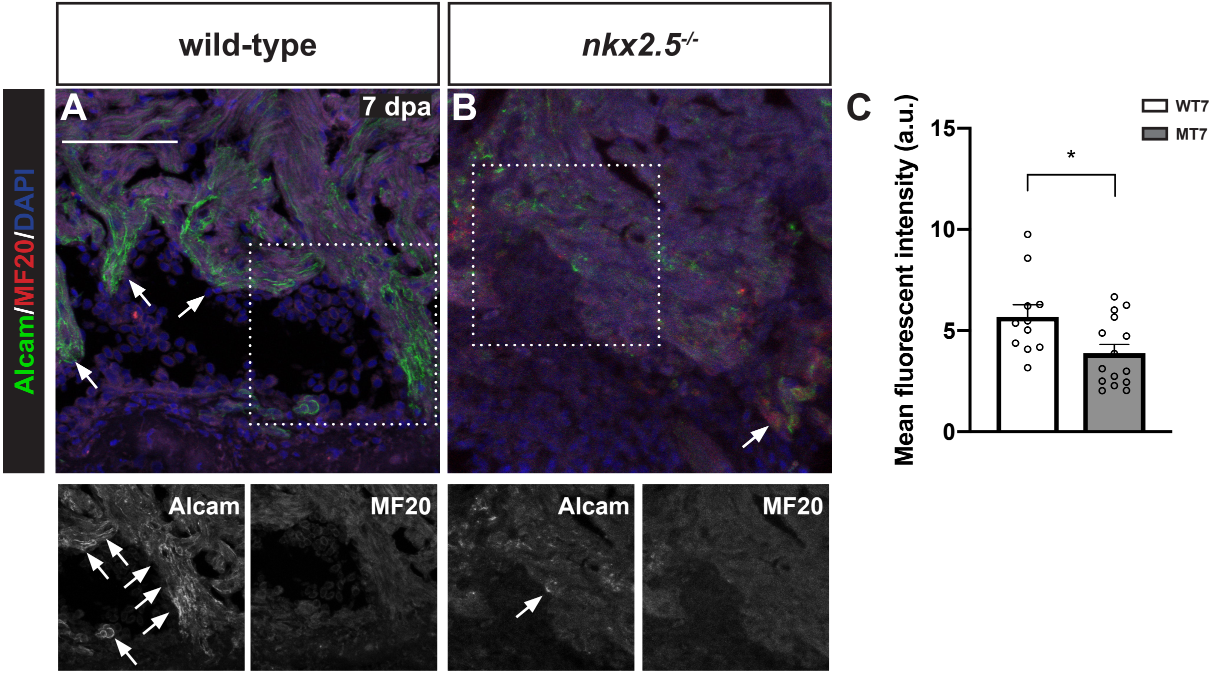

wild-type
nkx2.5-/-
Alcam/MF20/DAPI
A
7 dpa
B
C
Alcam
MF20
Alcam
MF20
WT7
MT7
Mean fluorescent intensity (a.u.)
15
10
5
0
*

Figure S10. Dedifferentiation is diminished in Nkx2.5 loss-of-function model

(**A,B**) Ventricular sections of non-transgenic wild-type (n = 3) (A) and *nkx2.5*$^{-/-}$ (n = 4) (B) were subjected to immunofluorescence with antibodies directed again Alcam and MF20 immunostaining. Insets represent single channel images of wound border delineated in the boxes. Scale bar, 50 μm.

(**C**) Quantification of integrated signal density of the Alcam stain is depicted. Mean and standard error of each data set are shown with an unpaired, two-tailed t-test illuminating a statistically significant difference between WT7 (n = 11) and MT7 (n = 15) (p = 0.0207).

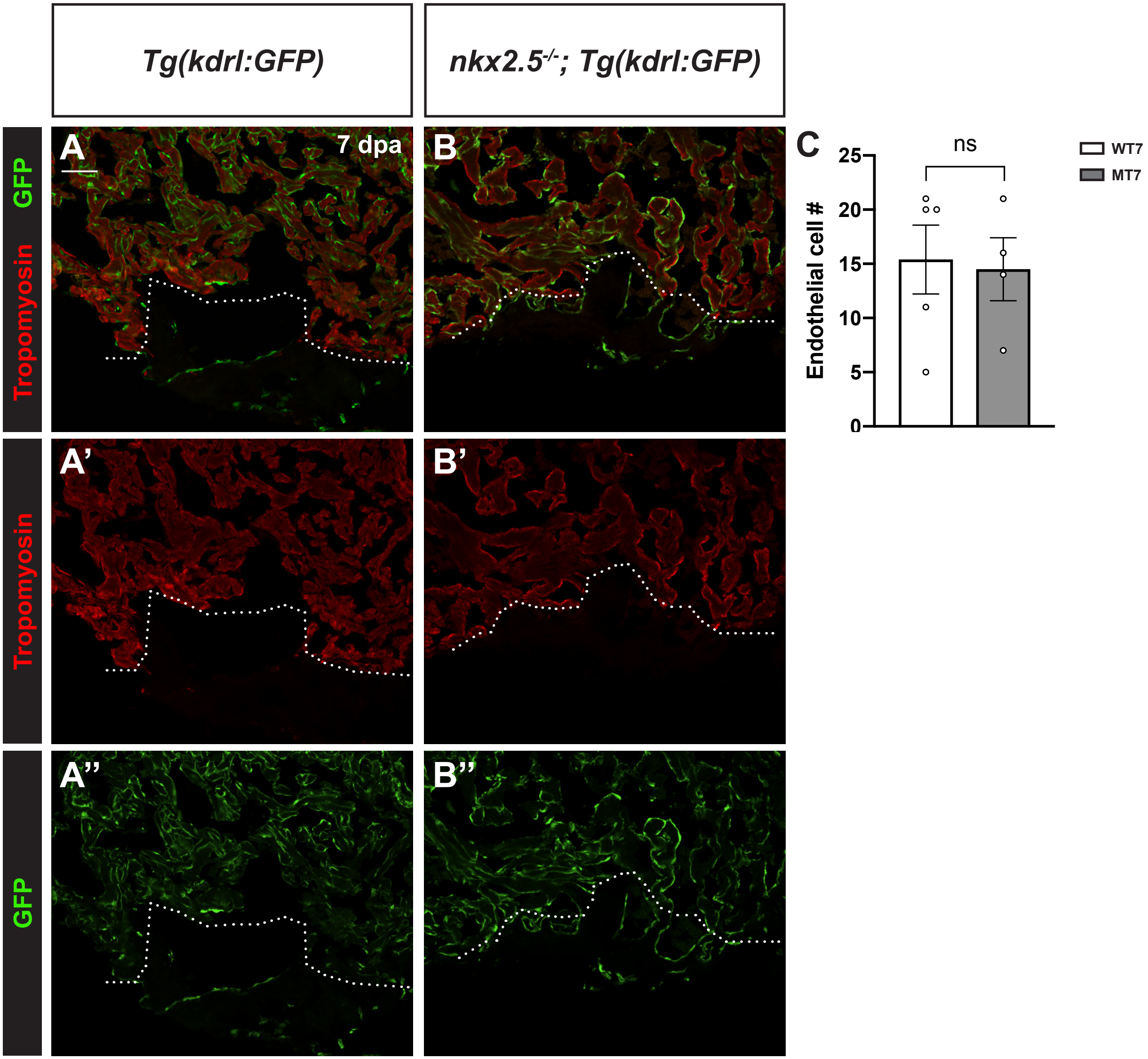

Tg(kdrl:GFP)
nkx2.5-/-; Tg(kdrl:GFP)
GFP
Tropomyosin
A
7 dpa
B
A'
B'
A''
B''
C
ns
WT7
MT7
Endothelial cell #
25
20
15
10
5
0

Figure S11. Angiogenesis is normal in resected *nkx2.5$^{-/-}$* ventricles

(**A-B''**) Tropomyosin immunostaining on sections of injured *Tg(kdrl:GFP)* (n =5) (A-A'') and *nkx2.5$^{-/-}$;Tg(hsp70l:nkx2.5-EGFP);Tg(kdrl:GFP)* (n = 4) (B-B'') hearts at 7 dpa showing normal endothelial tubes in the wound area. Scale bar, 50 μm.

(**C**) Quantification of endothelial cell number in *Tg(kdrl:GFP)* and *nkx2.5$^{-/-}$;Tg(hsp70l:nkx2.5-EGFP);Tg(kdrl:GFP)* fish reveals normal angiogenesis. Mean and standard error of each data set are shown with an unpaired, two-tailed t-test demonstrating no statistically significant difference (p = 0.8440) between WT7 (n = 5) and MT7 (n = 4).

Name	Sequence
cacna1c-forward	TCCGCTGCCAGTGAAATACT
cacna1c-reverse	TCCACGTCCACTCTCTGACA
col1a2-foward	AACAGCCGCTTCACTTTCAG
col1a2-reverse	CAATGTCCAAAGGTGCAATG
fn1a-forward	GAGCCTTTCACCCTTACAGC
fn1a-reverse	CCAGCCTCCTCATAGGTCAC
GFP-forward	CGACCACTACCAGCAGAACA
GFP-reverse	GAACTCCAGCAGGACCATGT
id2b-forward	GGACAGAGCCCTCCAAAAA
id2b-reverse	CGAGACAGGGCTATGAGGTC
mmp2-forward	AGCATGGGTCTTCCTTCAGA
mmp2-reverse	TCTAGGTCATCGGGAACAGC
mmp14a-forward	GCCATGCAGAAATTCTACGG
mmp14a-reverse	TTTATCAGGAACGCCACATCT
mmp14b-forward	TCCGAACTGAAGAGCAACCT
mmp14b-reverse	CGTAACGCTTTCCCACACTT
nfkbiaa-forward	CCATGGTGGAGAGTCTGGTC
nfkbiaa-reverse	CTGTGTATCCGCCGTAGGTT
pdlim5b-forward	GAACGCATGTGAGGTTGCTA
pdlim5b-reverse	ACATGGTTCACCTCCGTCTC
plk1-forward	CGGTGATAGCCTGCAGTACA
plk1-reverse	TAAGGCAATCGGGTCAGTTC
popdc2-forward	TTGATCCGGCACCATCTAAT
popdc2-reverse	ATCGAGGTGGAAAACGTCCT
postnb-forward	CAGAGTCCAGCATGTTCCAA
postnb-reverse	TCGGTAGGAGCGAAAAGTGT
tcap-forward	GAGAGTTACACCGCTGACTGG
tcap-reverse	CTGATGGAGAGGACGCACCT
vmhc-forward	AGGGAGGAAAGAGCATCCAT
vmhc-reverse	CTGGGCTCGCAGAATCTTAC

Supplemental Table 1. Primer sequences employed for quantitative PCR
