# Supplementary material for: Activation of Nkx2.5 transcriptional program is required for adult myocardial repair
Source: Nat Commun. 2022 May 27;13:2970. doi: 10.1038/s41467-022-30468-4 (PMC9142600; doi:10.1038/s41467-022-30468-4)
Supplement: Supplementary file 3 — Reporting Summary [file 41467_2022_30468_MOESM3_ESM.pdf]

nature portfolio

Corresponding author(s): Kimara L. Targoff

Last updated by author(s): Feb 9, 2022

Reporting Summary

Nature Portfolio wishes to improve the reproducibility of the work that we publish. This form provides structure for consistency and transparency in reporting. For further information on Nature Portfolio policies, see our Editorial Policies and the Editorial Policy Checklist.

Statistics

For all statistical analyses, confirm that the following items are present in the figure legend, table legend, main text, or Methods section.

n/a	Confirmed	
☐	☒	The exact sample size (*n*) for each experimental group/condition, given as a discrete number and unit of measurement
☐	☒	A statement on whether measurements were taken from distinct samples or whether the same sample was measured repeatedly
☐	☒	The statistical test(s) used AND whether they are one- or two-sided<br>*Only common tests should be described solely by name; describe more complex techniques in the Methods section.*
☒	☐	A description of all covariates tested
☒	☐	A description of any assumptions or corrections, such as tests of normality and adjustment for multiple comparisons
☐	☒	A full description of the statistical parameters including central tendency (e.g. means) or other basic estimates (e.g. regression coefficient) AND variation (e.g. standard deviation) or associated estimates of uncertainty (e.g. confidence intervals)
☐	☒	For null hypothesis testing, the test statistic (e.g. *F*, *t*, *r*) with confidence intervals, effect sizes, degrees of freedom and *P* value noted<br>*Give P values as exact values whenever suitable.*
☒	☐	For Bayesian analysis, information on the choice of priors and Markov chain Monte Carlo settings
☒	☐	For hierarchical and complex designs, identification of the appropriate level for tests and full reporting of outcomes
☒	☐	Estimates of effect sizes (e.g. Cohen's *d*, Pearson's *r*), indicating how they were calculated

Our web collection on statistics for biologists contains articles on many of the points above.

Software and code

Policy information about availability of computer code

Data collection

No data collection software packages were used in this study.

Data analysis

Images for AFOG, ISH, and RNAscope were acquired with a Zeiss Axio Imager.D2m and a Hamamatsu Nanozoomer SQ. Following immunostaining protocols, confocal imaging was performed with a Nikon Ti Eclipse inverted confocal microscope and a Zeiss LSM 880 with z-stacks analyzed using ImageJ (version 1.53k, NIH).

Reads were aligned to the GRCz10-91 (https://www.ncbi.nlm.nih.gov/assembly/GCF_000002035.5/) zebrafish reference genome using STAR (version 2.5.2) (Dobin et al., 2013) and were quantified using featureCounts (version 1.5.0-p3) (Liao et al., 2014). Differential gene expression between conditions was performed using DEseq2 (version 3.14) (Anders and Huber, 2012). Statistically significant differentially expressed genes (DEGs) were retrieved by filtering for genes with absolute log2 fold-change (FC) > 0.5 and false discovery rate (FDR) < 0.5 in R (version 3.6.0).

Venn overlaps of DEGs were generated with the VennDiagram package (version 1.7.1) (Chen and Boutros, 2011). Hierarchical clustering was performed using the gplots::heatmap.2() function which employs Euclidean distance measures with the average linkage method (version 3.1.1) (Warnes, 2020). The UpSet plot was generated with the UpSetR package (version1.4.0) (Conway et al., 2017). Functional enrichment was performed using Metascape 3.5 (Zhou et al., 2019) and validated by orthogonal approaches using DAVID (version 6.8) (Huang da et al., 2009) and Panther (version 14.1) (Mi et al., 2019), employing default parameters. Gene Ontology (GO) plots were created using the ggplot2 package (version 3.3.2).

Overlap with DamID targets of Nkx2-5 in mouse HL-1 cells (GSE44902) (Ramialison et al., 2017) was performed by first retrieving the zebrafish orthologues of the EnsEMBL IDs of the mouse DamID targets using BioMart (release 100) (Cunningham et al., 2019). Then, the intersection of the EnsEMBL IDs for the zebrafish DamID targets and zebrafish DEGs were obtained with BioVenn (version 1.1.3) (Hulsen et al., 2008). Network reconstructions were performed by retrieving protein-protein interacting relationships using STRING (version 11.0, default

parameters, https://string-db.org) and visualized with Cytoscape (version 3.8.0) (Shannon et al., 2003). For networks associated with GO terms, interactions only between genes associated with specified GO terms were examined. However, the networks highlighting DamID candidates as hubs (Fig. 6) were reconstructed based on an overlap of all targets and sets A and B (Fig. 5). The first neighbors of the DamID targets and modules with more than seven nodes were included for analysis.

For manuscripts utilizing custom algorithms or software that are central to the research but not yet described in published literature, software must be made available to editors and reviewers. We strongly encourage code deposition in a community repository (e.g. GitHub). See the Nature Portfolio guidelines for submitting code & software for further information.

Data

Policy information about availability of data

All manuscripts must include a data availability statement. This statement should provide the following information, where applicable:
- Accession codes, unique identifiers, or web links for publicly available datasets
- A description of any restrictions on data availability
- For clinical datasets or third party data, please ensure that the statement adheres to our policy

Previously published datasets employed in this manuscript include the zebrafish reference genome GRCz10-91 (https://www.ncbi.nlm.nih.gov/assembly/GCF_000002035.5/) and Nkx2-5 DamID targets (GSE44902). The RNA sequencing data newly reported in this paper is available under accession number GSE164966 at the Gene Expression Omnibus. Source data are provided with this paper. All other relevant data may be requested from the corresponding author.

Field-specific reporting

Please select the one below that is the best fit for your research. If you are not sure, read the appropriate sections before making your selection.

☒ Life sciences ☐ Behavioural & social sciences ☐ Ecological, evolutionary & environmental sciences

For a reference copy of the document with all sections, see nature.com/documents/nr-reporting-summary-flat.pdf

Life sciences study design

All studies must disclose on these points even when the disclosure is negative.

Sample size	We did not use statistical methods to determine sample size. The sample size follows common standards employing three or more biological replicates, consistent with similar publications in the field. Sample size is reported in the legends for all figures.
Data exclusions	Data was excluded from evaluation if there was evidence that the ventricular resection technique was not adequately performed.
Replication	We verified reproducibility of our studies by increasing the sample size whenever feasible. Furthermore, for quantitative measurements, two operators performed each analyses and their data was compared to demonstrate the ability to replicate our findings.
Randomization	Randomization was not applicable to this study. However, wild type and mutant fish were collected randomly for experiments with knowledge only of the presence or absence of the specific transgenes.
Blinding	For qualitative and quantitative measurements, both operators were blinded to the genotypes of each sample to ensure an unbiased assessment of the data.

Reporting for specific materials, systems and methods

We require information from authors about some types of materials, experimental systems and methods used in many studies. Here, indicate whether each material, system or method listed is relevant to your study. If you are not sure if a list item applies to your research, read the appropriate section before selecting a response.

Materials & experimental systems

n/a	Involved in the study	
☐	☒	Antibodies
☒	☐	Eukaryotic cell lines
☒	☐	Palaeontology and archaeology
☐	☒	Animals and other organisms
☒	☐	Human research participants
☒	☐	Clinical data
☒	☐	Dual use research of concern

Methods

n/a	Involved in the study	
☒	☐	ChIP-seq
☒	☐	Flow cytometry
☒	☐	MRI-based neuroimaging

Antibodies

Antibodies used	Primary antibodies used were: anti-PCNA (WH0005111M2, Sigma, 1:200), anti-Mef2 (sc-313, Santa Cruz Biotechnology 1:50), anti-tropomyosin (CH1, Developmental Studies Hybridoma Bank, 1:100), anti-myosin heavy chain (F59, Developmental Studies Hybridoma Bank, 1:25), anti-GFP (A-11122, Invitrogen, 1:200), anti-DsRed (632496, Clontech, 1:200), anti-raldh2 (GTX124302, GeneTex, 1:500), anti-vimentin (40E-C, Developmental Studies Hybridoma Bank, 1:35), anti-embCMHC (N2.261, Developmental Studies Hybridoma Bank, 1:50), MF20 (Developmental Studies Hybridoma Bank, 1:20), and anti-Alcam (ZN-8, Developmental Studies Hybridoma Bank, 1:10), and anti-DIG-AP antibody (11093274910, Roche; 1:2000). The following secondary antibodies were used (1:500): Alexa Fluor 488 Goat anti-Mouse IgG1 (A-21121, Invitrogen), Alexa Fluor 488 Goat anti-Mouse IgG2a (A-21131, Invitrogen), Alexa Fluor 568 Goat anti-Mouse IgG1 (A-21124, Invitrogen), Alexa Fluor 568 Goat anti-Mouse IgG2b (A-21144, Invitrogen), Alexa Fluor 568 Goat anti-Rabbit (A-11011, Invitrogen), Alexa Fluor 568 Goat anti-Mouse IgM (A-21043, Invitrogen), and Alexa Fluor 633 Goat anti-Rabbit (A-21070, Invitrogen).
Validation	All antibodies are commercially available and they have been tested by respective company. In detail: mouse anti-PCNA (Clone 1G7, Sigma WH0005111M2) has been validated by Sigma-Aldrich in immunoprecipitation, ELISA, immunofluorescence and wetern blot and detected in multiple species including zebrafish (see website). Rabbit anti-Mef2 (C-21, Santa Cruz sc-313) has been validated by Santa Cruz Biotechology in immunofluorescence and detected in different species (see website). Mouse anti-tropomyosin (CH1, Developmental Studies Hybridoma Bank (DSHB)) has been validated in immunoprecipitation, ELISA, immunofluorescence and western blot by DSHB and detected in multiple species (see website). Mouse anti-myosin heavy chain (F59, DSHB) has been validated in immunofluorescence, immunohistochemistry, immunoprecipitation and western blot by DSHB and detected in multiple species (see website). Rabbit anti-GFP (A-11122, Invitrogen) has been validated in western blot and immunohistochemistry by Invitrogen (see website). Rabbit anti-DsRed (632496, Clontech) has been validated for western blot and immunolabeling by Takara (see website). Rabbit anti-raldh2 (AB_11177627, GeneTex) has been validated in western blot and immunohistochemistry by GeneTex and detected in zebrafish (see website). Mouse anti-vimentin (40E-C, DSHB) has been validated in immunofluorescence and immunohistochemistry by DSHB and detected in multiple species (see website). Mouse anti-embryonic cardiac myosin heavy chain (N2.261, DSHB) has been validated in immunofluorescence, immunohistochemistry, ELISA and western blot by DSHB and detected in multiple species (see website). Mouse anti-Alcam (ZN-8, DSHB) has been validated in immunofluorescence and immunohistochemistry by DSHB and detected in zebrafish (see website). Mouse anti-myosin heavy chain (MF20, DSHB) has been validated in ELISA, immunofluorescence, immunohistochemistry, immunoprecipitation and western blot by DSHB and detected in multiple species (see website). The secondary antibodies Alexa Fluor 488 Goat anti-mouse IgG1, Alexa Fluor 488 goat anti-mouse IgG2a, Alexa Fluor 568 IgG1, Alexa Fluor 568 goat anti-mouse IgG2b, Alexa Fluor 568 goat anti-rabbit, Alexa Fluor 568 goat anti-mouse IgM and Alexa Fluor 633 goat anti-rabbit have been validated by Life Technologies (see website).

Animals and other organisms

Policy information about studies involving animals; ARRIVE guidelines recommended for reporting animal research

Laboratory animals	We used equal numbers of female and male adult zebrafish (3 months - 18 months) carrying the following previously described mutation and transgenes: nkx2.5vu179 (Targoff et al., 2013), Tg(hsp70l:nkx2.5-EGFP)fcu1 (George et al., 2015), Tg(nkx2.5:ZsYellow)fb7 (Zhou et al., 2011b), Tg(tcf21:DsRed2)pd37 (Kikuchi et al., 2011a), and Tg(kdrl:EGFP)la116 (Choi et al., 2007). Adult nkx2.5-/-;Tg(hsp70l:nkx2.5-EGFP) fish were generated as previously described (George et al., 2015) and experiments were implemented with one transgenic parent per cross.
Wild animals	The study did not involve wild animals.
Field-collected samples	The study did not involve field-collected samples.
Ethics oversight	All the zebrafish experiments were performed according to the protocol approved by the Institutional Animal Care and Use Committee (IACUC) at Columbia University.

Note that full information on the approval of the study protocol must also be provided in the manuscript.
